# Optimizing Immunohistochemistry Reporting in Endometrial Cancer

**DOI:** 10.7759/cureus.65810

**Published:** 2024-07-31

**Authors:** Rebecca Wood

**Affiliations:** 1 Histopathology, Royal Cornwall Hospitals Trust, Truro, GBR

**Keywords:** immunohistochemistry (ihc), pole mutation, endometrial ca, p53 endometrial carcinoma, endometrial cancer prognosis

## Abstract

The management of endometrial cancer involves a multidisciplinary team (MDT) approach, with immunohistochemistry playing an important role in management and prognosis. Markers investigated include estrogen receptor (ER), progesterone receptor (PR), tumor protein 53 (p53), and mismatch repair(MMR) protein. Additionally, polymerase epsilon (POLE) mutations indicate treatment-responsive tumors, often with excellent prognosis. We aimed to improve the reporting of immunohistochemistry and introduce POLE testing, with a sustainable change in the long-term management of endometrial cancer at Royal Cornwall Hospitals Trust (RCHT). An initial sample of 53 patients with endometrial cancer from 2022 was analyzed. Endometrial biopsy reports were reviewed for immunohistochemistry reporting, with delays of reporting over 10 days documented. In initial results, a mean of 15.5% of cases failed to report p53 (12/53), ER (9/53), PR (10/53), and MMR (2/53). The interventions implemented in February 2023 were an immunohistochemistry proforma, the introduction of POLE testing, and departmental presentations. Data was re-collected between March and September 2023. After the project, there was a 100% (39/39) rate of reporting immunohistochemistry correctly. POLE testing was introduced to the department. In addition, the proforma developed is now standard practice in the reporting of endometrial cancer cases and is utilized in the gynae-oncology MDT meetings.

## Introduction

Uterine cancer is the fourth most common cancer affecting women in the United Kingdom, with endometrium as the common specific location [1]. Histopathological assessment of endometrial biopsies is a key component in the diagnosis of endometrial cancer. Upon diagnosis, management is guided by multidisciplinary team (MDT) discussions and includes total hysterectomy, chemotherapy, and radiotherapy [2].

Immunohistochemistry plays a significant role in the management and prognosis of endometrial cancer [3]. Important immunohistochemistry markers include tumor protein 53 (p53), estrogen receptor (ER), progesterone receptor (PR), and mismatch repair (MMR) protein. Guidance from the British Association of Gynaecological Pathologists (BAGP) recommends that p53, ER, and MMR be tested in all cases of endometrial cancer [4]. P53 is a prognostic marker; tumors with aberrant p53 have a poorer prognosis and patients are more likely to receive neoadjuvant therapy [3]. ER and PR can differentiate between endometrioid and serous subtypes of endometrial cancer and determine hormone replacement options after surgery. MMR and methylation studies identify potential Lynch syndrome; if MMR is abnormal, treatment varies and can include immunotherapy. Additionally, tumors with DNA polymerase epsilon (POLE) mutations have a favorable prognosis and neoadjuvant treatment may not be indicated [5]. The cancer genome atlas is increasingly being used to stratify endometrial carcinomas into prognostic groups. P53 mutated cancers have the worst prognosis, MMR deficient cancers have an intermediate prognosis, and POLE mutation cancers have the best prognosis, with treatment being de-escalated in many cases [6]. Guidance from the BAGP recommends when to request POLE testing [4]. POLE testing is available for NHS patients via the national genomics service; however, this requires considerable resources and increases the workload in sample preparation and transport.

The aims of the project included optimizing the management of endometrial cancer by improving immunohistochemistry reporting at Royal Cornwall Hospitals Trust (RCHT). Specifically, we aimed to achieve 100% compliance with BAGP guidelines on immunohistochemistry reporting in endometrial cancer biopsies. In addition, we aimed to introduce POLE testing for certain cases of endometrial cancer: MMR deficient, p53-mutated, ER-negative, or stage 1b and above and to create a sustainable change in the long-term management of patients with endometrial cancer at RCHT. 

## Materials and methods

After an initial discussion with the pathology department, it was identified that immunohistochemistry reporting of endometrial cancer did not fulfill BAGP recommendations. In addition, reporting of endometrial cancer was variable between pathologists. Therefore, it was decided that a quality improvement project could be implemented. 

An initial sample of 53 patients with endometrial cancer from February 2022 to 2023 at RCHT was utilized. A biomedical scientist working within the pathology department provided a sample of 65 patients diagnosed with endometrial cancer on biopsy within the last 12 months. 53 patients were identified, utilizing inclusion and exclusion criteria. Inclusion criteria consisted of newly diagnosed endometrial carcinoma on biopsy. Exclusion criteria consisted of patients with additional cancer diagnosis on biopsy, such as ovarian, or endometrial hyperplasia. Endometrial biopsy reports were reviewed retrospectively for immunohistochemistry reporting by a single clinician. Immunohistochemistry reports were reviewed for reporting of p53, ER, PR, MMR, and Ki67. Interventions were implemented in February 2023, and thereafter data was re-collected from a second sample of newly diagnosed endometrial cancer cases. Thirty-nine cases of endometrial cancer between March and September 2023 were utilized.

**Figure 1:**
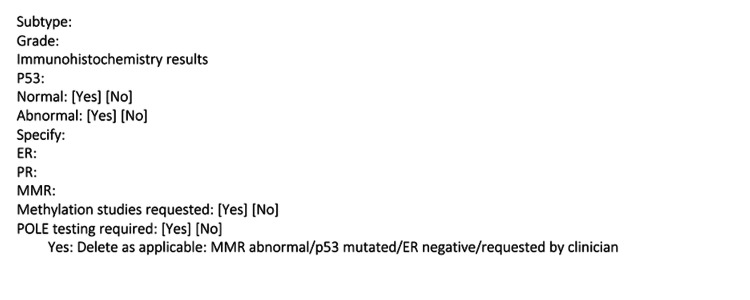
Proforma for endometrial carcinoma on biopsy specimens

Interventions were implemented after cycle one of data collection and included departmental presentations, implementation of a proforma, and introduction of POLE testing. Departmental presentations were carried out within the histopathology team, alongside the gynae-oncology MDT meetings. This aimed to highlight the clinical significance of correct immunohistochemistry reporting as well as introducing POLE testing. The proforma implemented aimed to standardize reporting of endometrial cancer while acting as a prompt for reporting (Figure 1). The proforma was included in the department’s shared folder for access, with a computer shortcut created for quick access to the proforma. Clinicians could utilize the proforma in the initial reporting of endometrial biopsies with carcinoma and within the gynae-oncology MDT. 

## Results

**Figure 2:**
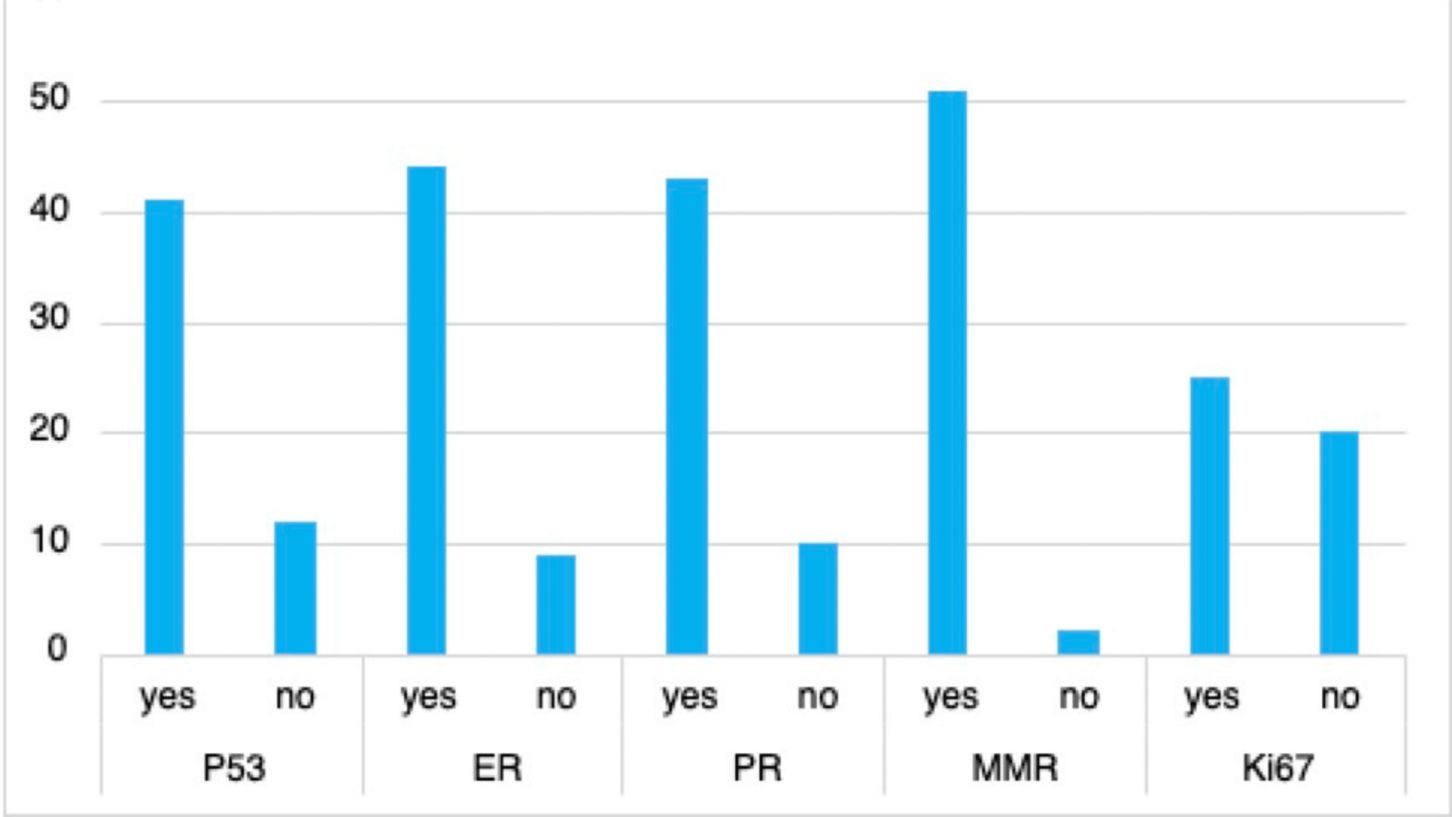
Pre-intervention immunohistochemistry reporting ER, estrogen receptor; PR, progesterone receptor; p53, tumor protein 53; MMR, mismatch repair

Initial data collection identified sub-optimal reporting of immunohistochemistry, with a mean of 15.5% failing to comply with reporting of p53 (12/53), ER (9/53), PR (10/53), and MMR (2/53) (Figure 2). The reporting of Ki67 was highly variable between pathologists. There were delays in immunohistochemistry reporting over 10 days in 34% (18/53) of cases. Delays were documented if an addendum of further immunohistochemistry had been added to the initial report, with a threshold of 10 days since initial reporting. 

**Figure 3:**
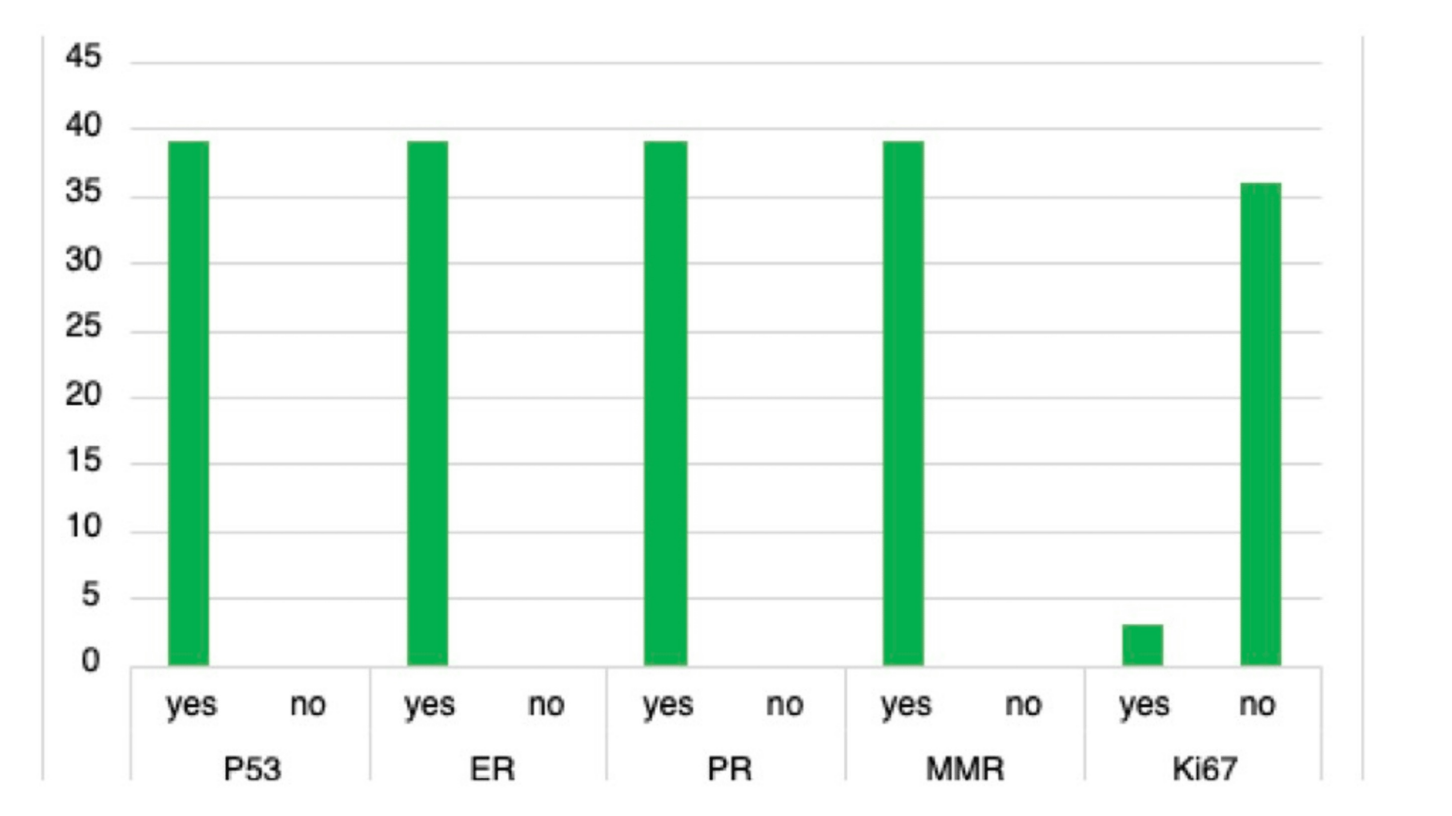
Post-intervention immunohistochemistry reporting ER, estrogen receptor; PR, progesterone receptor; p53, tumor protein 53; MMR, mismatch repair

Post-intervention data was recollected from a second sample, revealing improvement (Figure 3). 100% (39/39) of endometrial cancer cases reported p53, ER, PR, and MMR. Ki67 was no longer reported after discussions within the departments revealed minimal clinical significance. 62% (24/39) of cases utilized our new POLE proforma, and when completed, 63% (15/24) of those cases were sent to Bristol for testing. Delays in reporting remained similar with 44% (17/39) of cases having delays over 10 days. 

## Discussion

Initially, departmental presentations highlighted the importance of immunohistochemistry reporting in the management of endometrial cancer, alongside initial findings [4]. Presentations were delivered to the histopathology department, alongside the gynae-oncology MDT meeting. During the gynae-oncology meeting, it was identified that the current panel of immunohistochemistry included Ki67, which neither gynecology nor oncology utilized. This was subsequently removed from the department’s immunohistochemistry panel. Research suggests that Ki67, a marker of cellular proliferation, could indicate cancer-specific survival, with higher Ki67 scores indicative of reduced survival. One paper examined the use of hot-spot scoring to measure Ki67, suggesting this creates a clinically relevant marker. However, there are currently no guidelines to standardize measurement, and thus its clinical relevance requires further research [7]. 

The language surrounding the immunohistochemistry reporting was discussed, highlighting the confusion around the terminology of p53, which can be referred to as "positive/negative," "wild type," "aberrant," and "normal/abnormal." Therefore, it was agreed that p53 would be clearly documented as normal or abnormal for mutual understanding between teams. BAGP guidelines further emphasize the importance of clear reporting of p53, stating that p53 should be reported as normal or abnormal [4]. Reporting as positive or negative should be avoided. 

Additionally, POLE testing was introduced to the department. The gynecologists and oncologists were enthusiastic about the introduction of POLE testing, due to the clinical significance of POLE-positive endometrial cancer. A retrospective cohort study identified that women with POLE mutated endometrial cancer had excellent outcomes, suggesting that further research is required to determine if treatment is indicated in this group of women [8]. Further research has provided guidance on the classification of POLE-mutated endometrial cancer, thus facilitating its use in clinical practice [9]. Although POLE testing is recommended by the World Health Organisation, it is appreciated that testing requires significant resources and increases the workload for histopathology departments [4]. Nonetheless, the RCHT histopathology department recognized the importance of adding POLE testing as standard practice within new cases of endometrial cancer. Due to limited resources, the department utilizes POLE testing in specific circumstances as guided by BAGP: MMR deficient, p53 aberrant, ER-negative, or stage 1b. 

Finally, proforma implementation clearly outlined the required immunohistochemistry and prompted clinicians to consider POLE testing. A shortcut was created on departmental computers for easy accessibility to the proforma when reporting endometrial biopsy samples. During gynae-oncology MDT, the proforma could prompt communication of immunohistochemistry. Research on different types of cancer has suggested that proforma implementation could improve histopathology reporting [10]. Our results did indicate delays over 10 days with certain reporting of immunohistochemistry. This could be partly indicative of the high-volume workload pathology departments receive. Furthermore, consideration must be given to whether samples require transfer to national locations for molecular testing. Within Cornwall, methylation and POLE testing require samples to be sent to Bristol, which prolongs the full reporting of samples. Nonetheless, this project suggests a positive association between the implementation of a proforma and the correct reporting of immunohistochemistry. This project focuses on short-term impact; re-auditing the data could provide insight into the long-term impact and sustainability of the proforma. 

Although this project suggests a positive outcome, it is important to recognize limitations. First, our sample size was small, which reduced the generalizability of the results. This project was conducted in a district general hospital, thus limiting sample sizes and generalizability to alternate settings. Nonetheless, research within different cancers and institutions has suggested improved reporting of immunohistochemistry with proforma use [10]. In addition, quality improvement projects are often retrospective in nature and lack control groups that could introduce bias and prevent direct comparison of outcomes. However, this project aimed to focus on process improvement of immunohistochemistry reporting in endometrial cancer, rather than statistical inference. 

## Conclusions

At the conclusion of the project, there was a 100% (39/39) rate of reporting immunohistochemistry correctly. The introduction of a proforma was an easily accessible intervention with associated improvement in immunohistochemistry reporting. Interventions initiated the consideration of POLE testing within RCHT. Utilizing a proforma could help pathologists manage high workloads while maintaining a sustainable change in immunohistochemistry reporting. It is important to recognize that national guidance on immunohistochemistry reporting can vary between local departments. Quality improvement projects could prompt consideration of up-to-date guidance while introducing interventions associated with improved immunohistochemistry reporting in endometrial cancer.
